# Levels of Biological Markers of Nitric Oxide in Serum of Patients with Mandible Fractures

**DOI:** 10.3390/jcm10132832

**Published:** 2021-06-26

**Authors:** Lukasz Wozniak, Wioletta Ratajczak-Wrona, Jan Borys, Bozena Antonowicz, Karolina Nowak, Piotr Bortnik, Ewa Jablonska

**Affiliations:** 1 Department of Dental Surgery, Medical University of Bialystok, 15-276 Bialystok, Poland; lukasz.wozniak@umb.edu.pl (L.W.); bozena.antonowicz@umb.edu.pl (B.A.); 2 Department of Immunology, Medical University of Bialystok, Jerzego Waszyngtona 15A, 15-269 Bialystok, Poland; karolina.nowak@umb.edu.pl (K.N.); ewa.jablonska@umb.edu.pl (E.J.); 3 Department of Maxillofacial and Plastic Surgery, Medical University of Bialystok, 15-276 Bialystok, Poland; jan.borys@umb.edu.pl (J.B.); czaves82@wp.pl (P.B.)

**Keywords:** nitric oxide (NO), malonyldialdehyde (MDA), nitrotyrosine (NT), asymmetric dimethylarginine (ADMA), mandibular fractures

## Abstract

Background: Nitric oxide is a small gaseous molecule with significant bioactivity. It has been observed that NO may have a dual role dependent on its production and concentrations in the bone microenvironment. The objective of the study was to assess the concentration of total nitric oxide malonyldialdehyde, nitrotyrosine, and asymmetric dimethylarginine in the serum of patients with mandibular fractures and to understand the relationship between these compounds, in order to expand the knowledge base of the role of nitric oxide and its activity indicators in the process of bone fracture healing. Material and Methods: The study included 20 patients with mandibular fractures who were undergoing inpatient and outpatient treatments and a control group of 15 healthy people. Results were analyzed with respect to the measurement time. Total nitric oxide concentration in the blood serum was determined according to the Griess reaction, while the concentration of malonyldialdehyde, nitrotyrosine, and asymmetric dimethylarginine was estimated using the immunoenzymatic method (i.e., enzyme-linked immunosorbent assay). Results: Before the procedure, as well as on the first day and 2 and 6 weeks after the procedure, higher concentrations of total nitric oxide and lower concentrations of malonyldialdehyde were observed in the blood serum of patients with mandibular fractures compared to the control group. No statistically significant differences were found in nitrotyrosine concentrations in the blood serum of patients throughout the measurement period. However, a significantly higher asymmetric dimethylarginine concentration was observed in the patient serum before the procedure and on the first day of operation as compared with the control group. Analysis of the results observed in patient serum with respect to the number of fractures within the mandible demonstrated the same trend of concentrations for the tested compounds for the entire study group. Conclusions: In summary, our results revealed that the intensity of local processes resulting from mandibular fractures is associated with the concentration of nitric oxide, confirming its significant role, as well as that of its indicators, in the process of bone fracture healing in this patient population.

## 1. Introduction

Each tissue injury, even a very small one, induces a systemic reaction in the form of inflammation. Following an injury, numerous antigens reach the extracellular space penetrating from the outer environment, as well as from the inside of damaged cells. These damaged endothelial cells, as well as the lymphocytes and macrophages that are present near the injury site, start releasing different proinflammatory mediators, including numerous cytokines, which in turn stimulate the cells of the immune system to produce considerable amounts of nitric oxide (NO) through prolonged activation of inducible nitric oxide synthase (iNOS). The iNOS enzyme catalyzes the transformation of L-arginine (amino acid) into NO and citrulline [1,2,3].

NO is a molecule that regulates numerous physiological processes, including the activity of bone-forming cells. The normal course of the processes of bone formation and resorption constitutes bone remodeling and maintenance of the correct balance between the actions of osteoblasts and osteoclasts, thus enabling the preservation of bone robustness and repairing of microscopic lesions within the bone [4,5].

The bone healing process involves a rapid chain of cellular and biochemical reactions, initiated by an injury, the objective of which is to restore the continuity and primary structure of the damaged bone [6,7].

Two basic mechanisms take place for restoring the continuity of osseous tissue: spontaneous fracture healing (callus) and primary fracture healing. Spontaneous fracture healing occurs under the conditions of poor mobility of the fractured elements and enables their micromovements. A characteristic feature of this healing mechanism is that it is distinguished into the following phases: inflammatory (0–7 days), granular (8–14 days), callus formation (1–4 months), and callus remodeling (1–4 years). These phases reflect the significant morphological and biochemical changes that occur within and around the fracture site [7,8,9,10].

The influence of injuries, including those resulting from an operative procedure, on the immune system has been a subject of interest for a considerable period of time. Clinical studies have shown an increased concentration of the C3 complement component in patients after singular injuries, which has been linked with the dysfunction of multiple systems. In addition, it has been reported that the activity of natural killer cells is inhibited in patients after operative procedures. Moreover, individual studies have demonstrated functional disorder of phagocytic cells, dysregulation of chemotaxis, and inhibition of the antibacterial activity of neutrophils in patients who had experienced significant operational trauma or singular injuries (in the head or locomotor system) [11,12,13].

It appears that the concentration at which NO acts on bone cells is of key importance as it determines the influence of this compound on the processes associated with the functioning of these structures. On the one hand, researchers have reported that constitutively secreted NO, together with endothelial nitric oxide synthase (eNOS), is a prerequisite for the normal functioning of osteoblasts and that it has a positive impact on injury healing processes. On the other hand, some publications in the literature highlight the negative influence of high NO concentrations, which inhibit the growth and differentiation of osteoblasts, even leading to the loss of bone mass. Although the mechanism of action of NO on bone cells is unknown, it can be expected that it plays a significant role in bone metabolism [5,14,15,16,17].

NO metabolism includes the formation of numerous intermediate products that exhibit high reactivity, among which nitrogen occurs at different oxidation states (ranging from +1 to +5). The final products of NO metabolism are nitrites (NO_2_^−^) and nitrates (NO_3_^−^). Peroxynitrite, which is a product resulting from the reaction between NO and a superoxide anion radical, is attributed with considerable significance in the mechanisms of action of NO. A series of (relatively) stable potential markers for the formation of reactive nitrogen species in vivo have been identified, including malonyldialdehyde (MDA) and nitrotyrosine (NT). In addition, there already exists a known indicator of the production and bioavailability of NO—asymmetric dimethylarginine (ADMA) [18,19].

**Figure 1:**
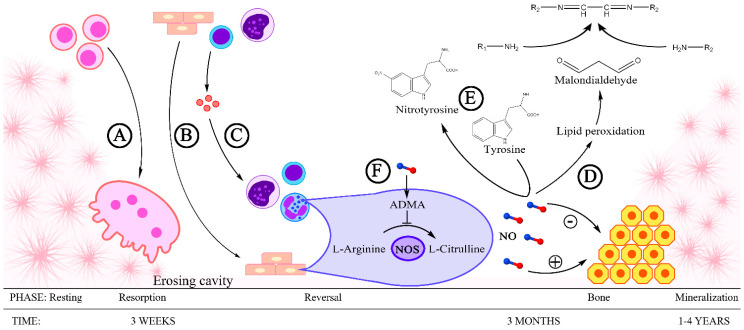
Nitric oxide and its activity indicators in the process of bone fracture healing: A—Preosteoclasts fuse to form osteoclasts, the cells that participate in the resorption of bone tissue. B—In endothelial cells, monocytes, and lymphocytes, the expression of endothelial nitric oxide synthase is increased, which is demonstrated by the production of nitric oxide (NO). NO intensifies the process of bone formation from osteoblasts. C—Endothelial cells, monocytes, and lymphocytes constitute a source of proinflammatory cytokines, which activate neutrophils and macrophages. The expression of inducible nitric oxide synthase is intensified in these cells, leading to the generation of large amounts of NO. High NO concentration has an inhibitory effect on the process of osteoblast differentiation and bone formation. D—NO intensifies lipid peroxidation, as a result of which malonyldialdehyde is produced, which participates in the formation of aminoiminopropene Schiff bases. E—NO is one of the factors that nitrate the tyrosine phenolic units leading to the formation of nitrotyrosine. F—ADMA, an endogenous inhibitor of eNOS.

The present study aimed at assessing the concentration of total NO, MDA, NT, and ADMA in the blood serum of patients with mandibular fractures and understanding the relationships between these compounds, in order to expand the knowledge base of the role of NO and its activity indicators in the process of bone fracture healing (Figure 1).

## 2. Materials and Methods

The study included 20 male patients aged 18–49 (mean 27.25) years, who were hospitalized due to mandibular fractures at the Department of Maxillofacial and Plastic Surgery of the Medical University of Bialystok.

The control group was comprised of 15 healthy individuals (men), who were voluntary blood donors, aged 20–30 years, and had no systemic diseases and bone fractures within the last 5 years. Blood was collected from them after obtaining written approval for blood donation.

Blood parameters of control group and patients were presented in Table 1. No difference between those groups were observed.

All individuals were informed of the study methodology, after which they provided written consent to participate in the experiments. The study was approved by the Ethics Committee of the Medical University of Bialystok (R-I-002/394/2016). Among the patients, 15 (75%) suffered from mandibular body fracture, 1 (5%) had a fracture within the area of mandibular processes, and 4 (20%) had a fracture in the mandibular body and processes. Eight patients (35%) had a singular mandibular fracture, and 12 (60%) had double mandibular fractures. All of the experiments were performed in accordance with good laboratory practice.

The injuries were primarily caused by beating (13 patients, 65%) and falls (5 patients, 25%). One person (5%) was injured in a car accident, while in 1 (5%), the fracture cause could not be identified.

**Figure 2:**
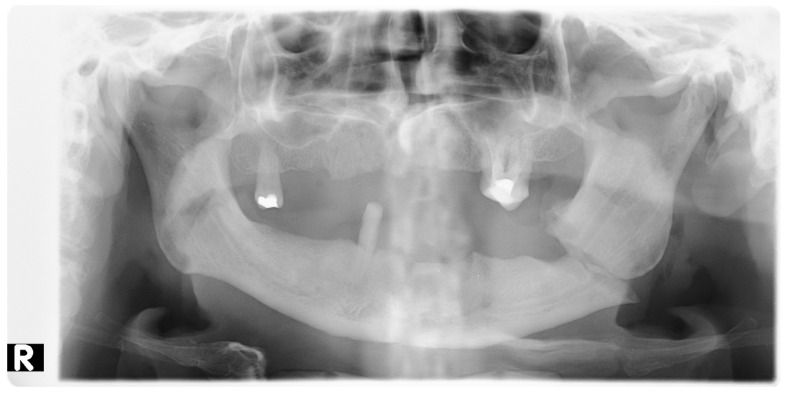
Pantomographic image showing interrupted continuity of the bone structure of the mandibular body near the angle of the mandible, on the left side, with splinter displacement. R—right side.

**Figure 3:**
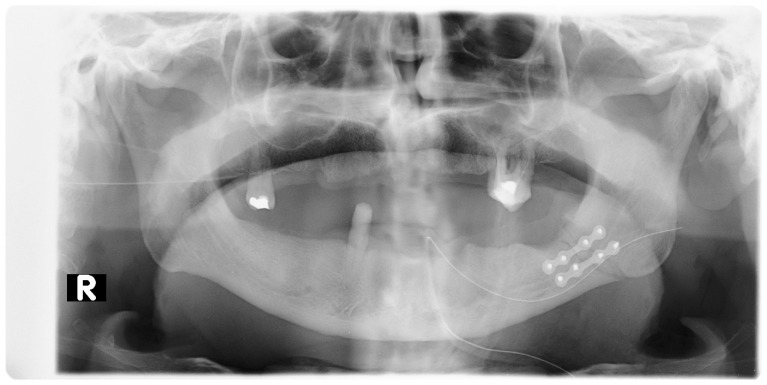
Pantomographic image showing mandible miniplate osteosyntheses with screws: 2 miniplates with 8 screws near the angle of the mandible, left side. R—right side.

**Figure 4:**
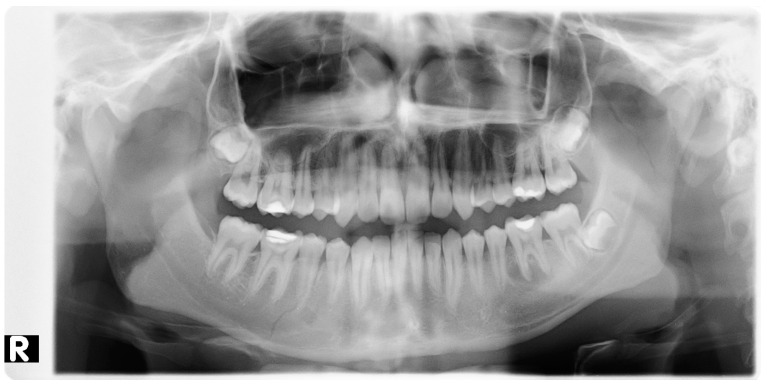
Pantomographic image showing an interrupted bone structure without splinter displacement near tooth 45 and near the mandibular condyle on the right side, with low fracture type. R—right side.

**Figure 5:**
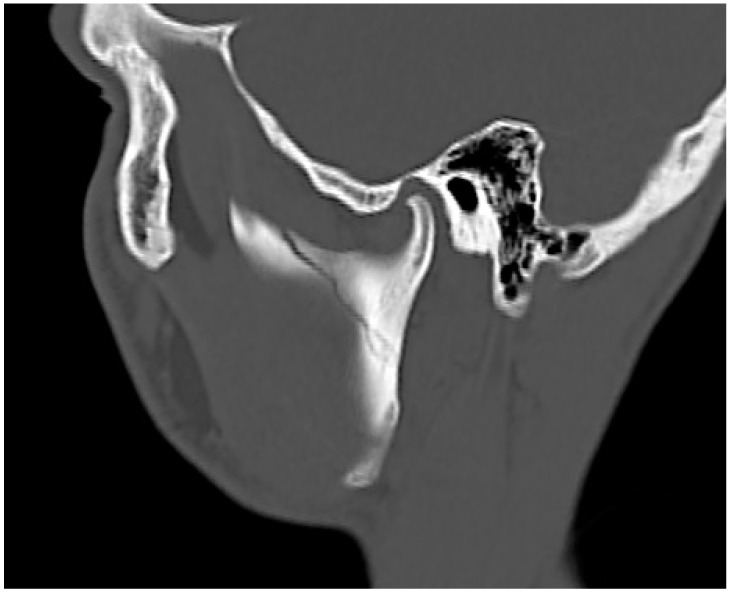
Computed tomography showing the presence of a linear fracture gap at the base of the left mandibular condyle without splinter displacement.

**Figure 6:**
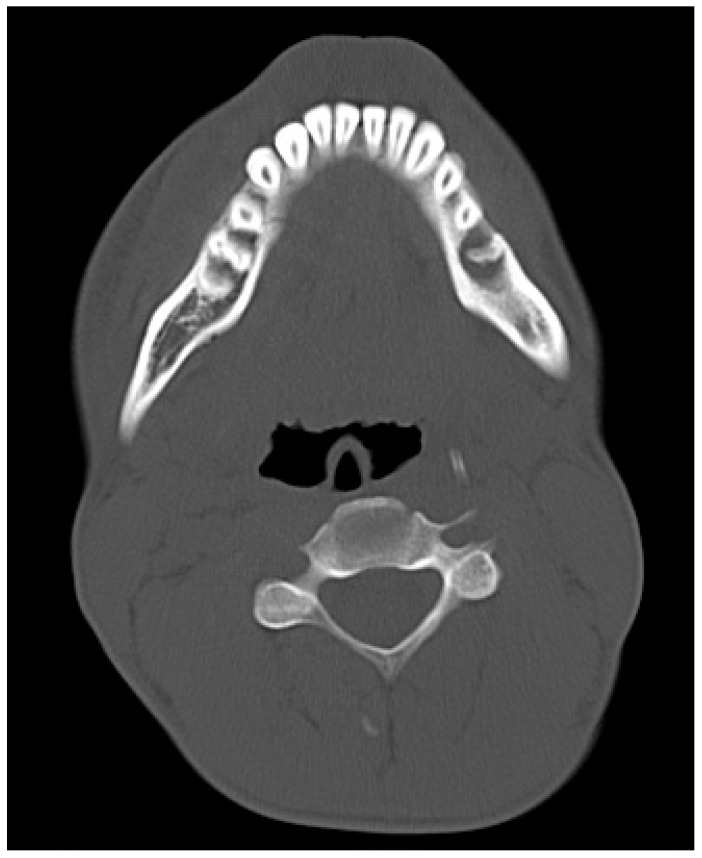
Computed tomography in axial plane showing the interrupted continuity of the bone structure near tooth 45.

**Figure 7:**
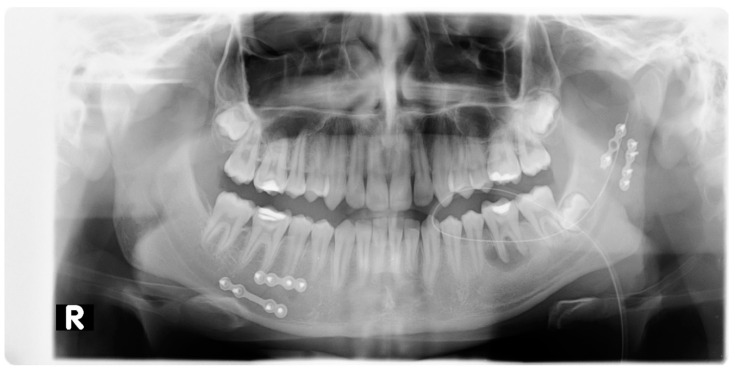
Pantomographic image showing mandible miniplate osteosyntheses with screws: 2 miniplates with 8 screws near tooth 45 and 2 miniplates with 6 screws at the base of the mandibular condyle. R—right side

**Figure 8:**
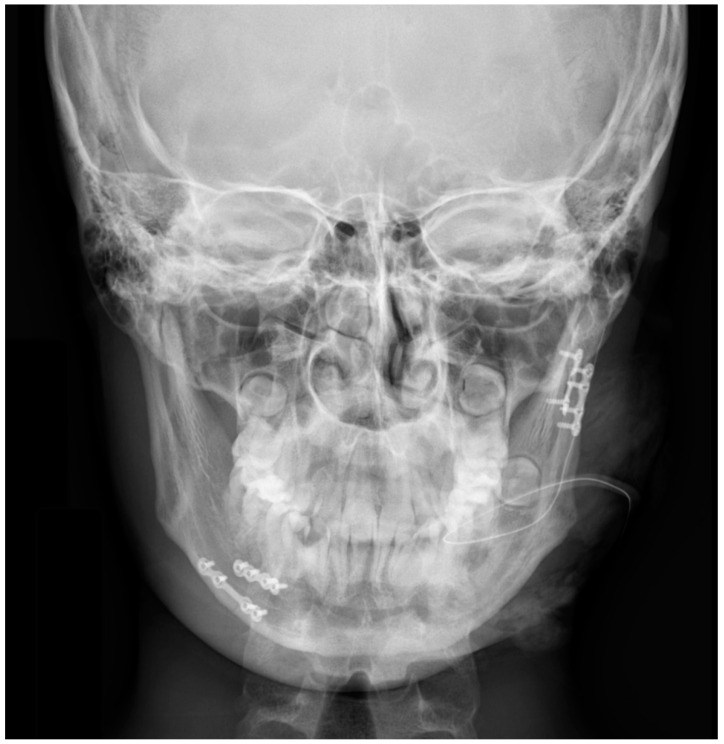
Posterior–anterior image of the mandible showing the presence of plate anastomosis of the mandible with screws: 2 miniplates with 8 screws near tooth 45 and 2 miniplates with 6 screws at the base of the mandibular condyle.

All patients (100%) included in the study were otherwise healthy (Table 1). Eleven (55%) declared that they smoked cigarettes on a regular basis. All the patients were subjected to examinations such as pantomographic X-ray (Figure 2, Figure 3 and Figure 4, computed tomography (Figure 5 and Figure 6), pantomographic X-ray (Figure 7), mandible X-ray in posterior–anterior projection (Figure 8), blood morphology and coagulation testing, analysis of electrolyte levels (Table 1), and electrocardiography. One day before the procedure, they were administered an antibiotic (cefazolin, 2 × 1.0 g iv) and a proton pump inhibitor with stomach-protective function (esomeprazole, 1 × 40 mg iv). All of the patients were operated on about 4 days from the injury under general endotracheal anesthesia. As a part of premedication, they were administered 7.5 mg midazolam approximately 40 min before the procedure. Mandibular fractures were dressed through open reposition and osteosynthesis of the fragments via miniplates and titanium screws. Eleven patients (55%) had teeth remaining in the fracture gap. On the day of the procedure, the antibiotic and gastroprotective drug were again administered to the patients. In addition, an analgesic was introduced (ketoprophen, 3 × 100 mg iv). A day after the procedure, the patients received 1 dose of dexamethasone—4 mg iv, and 2 days after the procedure, 1 dose of dexamethasone—2 mg iv. The antibiotic, gastroprotective drug, and analgesic (vitamin C, 3 × 300 mg) continued to be administered to the patients throughout their stay at the hospital. All of the patients had intermaxillary tractions installed on the MMF (maxillomandibular fixation) screws during the procedure. The tractions were kept for a period of about 2 weeks.

After hospitalization, the patients were recommended to ingest resorption drugs (calcium and vitamin C), and if needed, an analgesic (Ibuprofen or Paracetamol). In addition, the following were recommended: cold compresses to relieve swelling, Altacet compresses for 2–3 days, high-protein diet for 6 weeks, Eludril mouthwash thrice a day for 2 weeks, and dental check-ups. They were also advised to avoid excessive physical effort.

### 2.1. Blood Sampling

Patients with facial fractures and healthy control subjects were recruited into the study after obtaining their informed consent. Six milliliters of blood were collected by venous arm puncture under aseptic conditions into test tubes with clot activator. Blood was collected at four time points: before surgery (T0), at 24 h after surgery (T1), at 2 weeks (T2), and at 6 weeks (T3) after surgery. Serum was obtained by centrifugation at 2000 rpm for 10 min of blood samples and freeze at −80 °C until the date of analysis.

### 2.2. Determination of Total NO Concentration in Serum

Nitrite (NO_2_^−^) and nitrate (NO_3_^−^) are stable final products of NO metabolism and may be used as indirect markers of NO. Total NO concentration was determined as sum of NO_2_^−^ and NO_3_^−^ concentrations in indirect method based on measurement of NO_2_^−^ concentration in serum according to Griess reaction. In the presence of cadmium (Sigma-Aldrich, Steinheim, Germany), NO_3_^−^ was reduced to NO_2_^−^, which is able to react in a colorful reaction with Griess’s reagent (Sigma-Aldrich, Steinheim, Germany). Nitrite concentrations were determined by spectrophotometric analysis at 540 nm (UVN-340 ASYS Hitech GmbH microplate reader; Biogenet, Eugendorf, Austria) with reference to a standard curve. NO products were expressed as μM.

### 2.3. Determination of Malonodialdehyde (MDA) Concentration in Serum

The concentration of malonodialdehyde (MDA) in serum was assessed by quantitative competitive enzyme-linked immunosorbent assay (ELISA) using a commercially available kit (Human Malonodialdehyde (MDA) ELISA kit; MyBioSource, San Diego, USA). Prior to the experiment, all provided reagents were held in room temperature for 30 min. Standards (100 µL), controls (100 µL), and serum samples (100 µL) were pipetted to anti-MDA antibody-coated wells. Then horseradish peroxidase (HRP)-conjugate (50 µL) was added and a 96-well plate was incubated for 60 min at 37 °C. After washing 5 times, substrates for HRP enzymes were added (substrate A and B; 50 µL each). After 20 min incubation at 37 °C, stop solution was added to terminate the reaction, which turned the color from blue to yellow. The intensity of color was measured spectrophotometrically at 450 nm with a ELx800 Absorbance Reader (BioTek Instruments, Winoosky, Vermont, USA). The MDA concentration was evaluated based on the standard curve. The obtained results were expressed as ng/mL.

### 2.4. Determination of Nitrotyrosine Concentration in Serum

The concentration of nitrotyrosine in serum was assessed by quantitative sandwich enzyme-linked immunosorbent assay (ELISA) using a commercially available kit (Nitrotyrosin ELISA Kit; Immunodiagnostic AG, Bensheim, Germany). Prior to the experiment, all provided reagents were held in room temperature for 30 min. Standards (100 µL), controls (100 µL) and serum samples (diluted 1:60 in assay buffer; 100 µL) were added to polyclonal goat anti-nitrotyrosine antibody-coated wells. The plate was incubated for 60 min at 37 °C and then washed 5 times. Then a peroxidase-conjugated polyclonal goat anti-human serum proteins antibody (100 µL) to the wells and the plate was incubated for 60 min in a microplate shaker. After washing 5 times, the substrate (tetramethylbenzidine, 100 µL) was added. Next, the plate was incubated in dark for 15 min and finally, an acidic stop solution (100 µL) was added to terminate the reaction. Absorbance of the colorful product was measured at 450 nm with a UVN-340 ASYS Hitech GmbH microplate reader (Biogenet, Eugendorf, Austria). The nitrotyrosine concentration was calculated from the standard curve of the absorbance vs. standard concentration. The obtained results were expressed as nM.

### 2.5. Determination of Asymmetrical Dimethylarginine (ADMA) Concentration in Serum

The concentration of asymmetrical dimethylarginine (ADMA) in serum was assessed by quantitative double-sandwich enzyme-linked immunosorbent assay (ELISA) using a commercially available kit (Human Asymmetrical Dimethylarginine (ADMA) ELISA Kit; MyBioSource, San Diego, CA, USA). Prior to the experiment, all provided reagents were held in room temperature for 30 min. Standards (50 µL), serum samples (50 µL), and sample diluent (50 µL) were added into a human ADMA monoclonal antibody-coated 96-well wells. Then the horseradish peroxidase (HRP)-conjugate reagent was added to each well and plate were incubated for 60 min at 37 °C. After washing 4 times, chromogen solutions A and B (50 µL each) were added to each well, then the plate was incubated in the dark for 15 min. Addition of stop solution (50 µL) terminated the reaction and changed sample color from blue to yellow. Absorbance was read at 450 nm with a UVN-340 ASYS Hitech GmbH microplate reader (Biogenet, Eugendorf, Austria). The ADMA concentration was calculated from the standard curve. The obtained results were expressed as ng/mL.

### 2.6. Statistical Evaluation

Data were analyzed using STATISTICA version 13.3 program (StatSoft, Inc., Tulsa, OK, USA) and results were express as mean ± standard deviation (SD). The categorical data from repeated measurements were analyzed by variance analysis. To assess the distribution of total nitric oxide, malonyldialdehyde, nitrotyrosine, and asymmetric dimethylarginine data Shapiro–Wilk’s test of normality and visual inspection of Q–Q plots was used. Data revealed a normal distribution, hence we used ANOVA with Tukey’s post hoc test and Student *t*-test for pairwise comparisons. The relationship between total nitric oxide and its activity indicators was analyzed by Spearman’s rank correlation test. The results were considered significant if *p-*values were 0.05 or less. The data were plotted using STATISTICA version 13.3 (STATSOFT; Statistica, Tulsa, OK, USA).

## 3. Results

Total NO concentration in patient serum.

**Figure 9:**
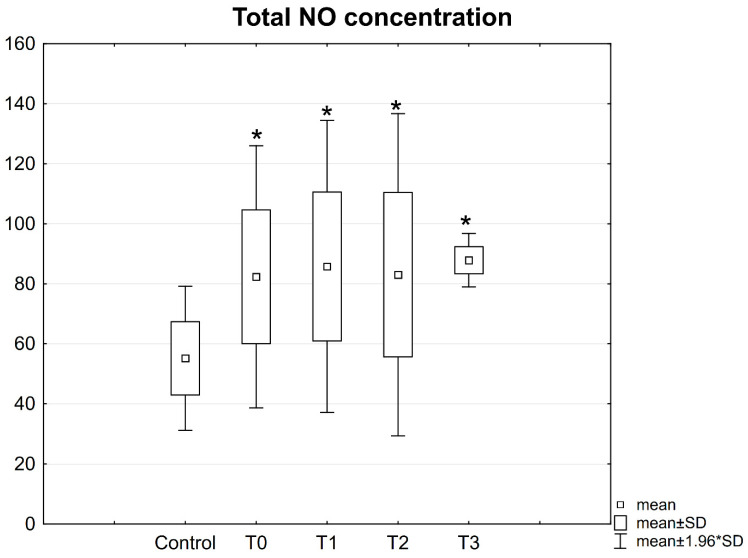
Concentrations of total NO in the serum of patients with mandible factures. All data are expressed as mean ± SD. Patients group: before surgery (T0), at 24 h after surgery (T1), at 2 weeks (T2), and 6 weeks (T3) after surgery. *—statistical differences with control.

Before the procedure, as well as on the first day and 2 and 6 weeks after the procedure, significantly higher concentrations of total NO were observed in the blood serum of patients with mandibular fractures compared to the control group (Figure 9).

However, no statistically significant differences were noted in the concentrations of total NO between the studied patient groups measured in different time checkpoints (Figure 9).

### 3.1. MDA Concentration in Patient Serum

**Figure 10:**
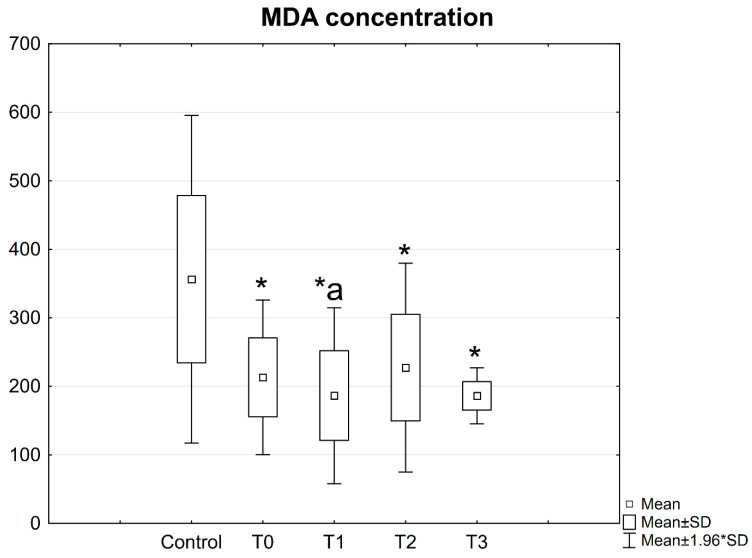
Concentrations of MDA in the serum of patients with mandible factures. All data are expressed as mean ± SD. Patients group: before surgery (T0), at 24 h after surgery (T1), at 2 weeks (T2), and 6 weeks (T3) after surgery. *—statistical differences with control; a—statistical differences between patients before surgery (T0) and at 24 h after surgery (T1).

Both before and after the operative procedure, a significantly lower concentration of MDA was observed in the patient serum as compared with the control group (Figure 10).

Moreover, the concentration of MDA was found to be lower (186.38 ng/mL) in the patient serum on the first day after the procedure compared to the values determined before the procedure (216.32 ng/mL; *p* = 0.031) (Figure 10).

### 3.2. NT Concentration in Patient Serum

**Figure 11:**
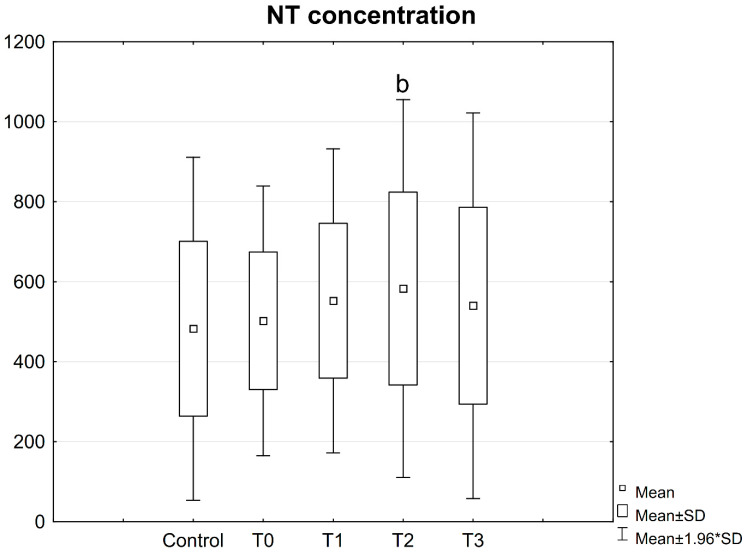
Concentrations of NT in the serum of patients with mandible factures. All data are expressed as mean ± SD. Patients group: before surgery (T0), at 24 h after surgery (T1), at 2 weeks (T2), and 6 weeks (T3) after surgery. b—statistical differences between patients before surgery (T0) and at 2 weeks after surgery (T2).

No statistically significant differences were observed in the concentration of NT in the blood serum of patients prior to as well as after the procedure in comparison with the control group (Figure 11).

However, higher NT concentrations were observed in the serum of patients 2 weeks after the procedure compared to the values determined before the procedure (Figure 11).

### 3.3. ADMA Concentration in Patient Serum

**Figure 12:**
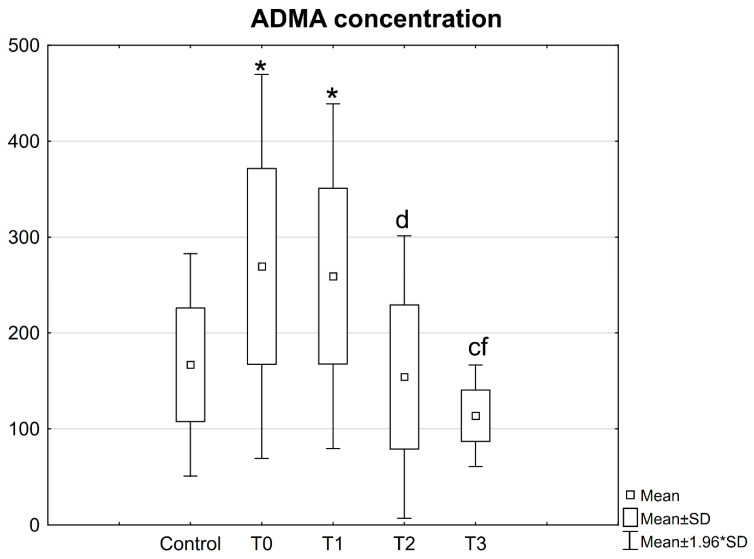
Concentrations of ADMA in the serum of patients with mandible factures. All data are expressed as mean ± SD. Patients group: before surgery (T0), at 24 h after surgery (T1), at 2 weeks (T2), and 6 weeks (T3) after surgery. *—statistical differences with control; c—statistical differences between patients before surgery (T0) and at 6 weeks after surgery (T3), d—statistical differences between patients at 24 h after surgery (T1) and at 2 weeks after surgery (T2); f—statistical differences between patients at 24 h after surgery (T1) and at 6 weeks after surgery (T3).

A significantly higher concentration of ADMA was observed in the serum of patients with mandibular fractures before and on the first day after the operation, as compared to the control group. The concentration of ADMA determined at these time points was also higher than the values obtained 6 weeks after the procedure (Figure 12).

Furthermore, the ADMA concentration estimated in patient serum on the first day after the procedure was found to be higher than the values estimated after 2 weeks (Figure 12).

### 3.4. Correlations

A positive correlation was found between the concentrations of total NO and ADMA in patient serum before the procedure (0.4975, *p* = 0.005).

In addition, a positive correlation was found between the concentrations of total NO and MDA in patient serum on the first day after the procedure (0.8133, *p* = 0.001).

### 3.5. Differences in Terms of the Number of Fractures 

The results observed in patient serum were also analyzed with respect to the number of fractures within the mandible (Table 2).

The concentration of total NO was found to be higher in the serum of all the patients with 1 or 2 fractures before and after the procedure, except for the patients with 2 fractures 2 weeks after the procedure. No differences were identified in the total NO concentrations in the serum between the patients with 1 and 2 fractures in the mandible.

Compared to the control group, the concentration of MDA was found to be lower in the serum of patients with 1 and 2 fractures (except for the patients with 1 fracture 2 weeks after the procedure). No differences were identified in NT concentrations in the serum between patients with 1 and 2 fractures in the mandible.

No statistically significant differences were found in NT concentrations in the serum of patients with 1 and 2 fractures as compared with the control group.

The concentration of ADMA was found to be higher in the serum before and 1 day after the procedure in patients with 1 and 2 fractures in comparison with the control group.

### 3.6. Correlations

A positive correlation was found between the concentrations of total NO and NT (0.9219, *p* < 0.05) and between the concentrations of NO and MDA (0.8902, *p* < 0.05) in the serum of patients with 1 mandibular fracture 1 day after the procedure.

In patients with 2 fractures, a positive correlation was found between the concentrations of total NO and ADMA in serum before the procedure (0.6194, *p* < 0.05) and between the concentrations of total NO and NT (0.6669, *p* < 0.05) and between the concentrations of MDA and NT (0.7336, *p* < 0.005) in the serum 1 day after the procedure.

## 4. Discussion

In normally functioning osseous tissue, osteoblasts and osteoclasts possess constitutively active eNOS, which produces low amounts of NO. The local synthesis of this molecule is sufficient to stimulate both these cell types. However, during inflammation, as a result of the action of inter alia, proinflammatory cytokines, the activity of iNOS may be restored along with an intensified production of NO. High concentrations of NO in the cytoplasm and intercellular space may lead to cell apoptosis, including that of pro-osteoclasts, as well as leukocyte deficiency, which contributes to an inhibited alteration of cytoskeleton [20,21,22]. Therefore, the high concentrations of total NO that were demonstrated in the serum of patients with mandibular fractures throughout the measurement period appear to be an alarming concern, particularly at the later stages of fracture healing.

The available data show that high concentrations of NO lead to the inhibition of osteoblast differentiation, which may result in a reduction in the number of these cells, and as a consequence, distressed reconstruction of the damaged tissue. On the other hand, prolonged exposure of osteoclasts to this molecule at a toxic concentration may stimulate them to resorb bone tissue, leading to the loss of bone mass and deconditioning of the mechanical properties of the skeleton [20,22,23,24,25].

Corbett et al. [26] demonstrated an elevated expression of eNOS in osteocytes and cortical bone vessels at the early stages of fracture (on the first day) as well as an elevated expression of iNOS in endosteal osteoblasts and chondroblasts at the later stages (from 2 weeks of injury) in a rabbit model. In addition, some studies have shown that eNOS may be activated via inter alia, an injury and a bone fracture, which would explain the high concentrations of total NO observed in the serum of patients, particularly on the first day of injury [4,17,21].

Similar observations were also reported by Keskin et al. [27], who demonstrated higher concentrations of total NO in the serum of patients with an isolated femur fracture, as well as in the serum of those with multiple fractures, including a femur fracture. However, in contrast to the results obtained in our study, the authors observed differences in the total NO concentrations in the serum of both patient groups depending on the measurement time—the highest values were found in the serum of patients at 6 h and after 14 days of fracture.

Prasad et al. [28] also demonstrated high NO concentrations in the serum of patients with an isolated femur fracture and those with other fractures in long bones apart from the femur. However, they noticed that the NO concentrations increased considerably on the 7th day, attaining the maximum on the 14th day and decreasing to normal on the 28th day.

Both studies by Keskin et al. [27] and Prasad et al. [28] showed higher concentrations of total NO in patients with multiple fractures than in those with single fractures. However, we could not identify statistically significant differences between patients depending on the number of fractures. It is surprising that we observed higher mean concentrations of total NO in the serum of patients with singular fractures than in those with two fractures. The fact that the results of the abovementioned authors are in contrast to those of the present study may be due to the expanse of the injury, which contributed to the activation of a greater number of cells.

The elevated production of NO in the blood serum may be responsible for its direct impact on the compounds that are essential for the cells of an organism. It has been demonstrated that NO plays a significant role in the process of lipid peroxidation. One of the numerous compounds that are produced during the peroxidation of polyunsaturated fatty acids is aldehydes, such as MDA [29,30,31]. Despite the presence of considerable amounts of NO in the serum of patients with fractures, we found the concentration of MDA to be low, which pointed out the increased total antioxidative capacity of the peripheral blood in these patients. On the other hand, the results observed for MDA may be a consequence of the administration of a strong antioxidant—vitamin C—to the patients throughout their stay at the hospital, as well as after hospitalization.

Different results were obtained by Göktürk et al. [32], who showed an increased concentration of MDA in the tibia of rats at 7 and 14 days after an experimental fracture. Furthermore, they suggested that the oxidative stress produced in these rats was responsible for the delayed fracture healing.

The results obtained in the aforementioned study indicate that fracture and normal course of healing of a relatively small bone, such as the mandible, result in changes in NO concentrations and the activity of the studied NO markers, similar to that in long bones.

NT is another significant marker of the elevated production of NO from NO metabolites. It is formed as a result of nitration of tyrosine phenolic units in tissues and proteins of the blood, contributing to increased susceptibility of proteins to the action of proteolytic enzymes. In addition, tyrosine nitration leads to the loss of biological functions of these proteins, and as a consequence, to pathological changes [33,34,35]. The high concentrations of total NO, accompanied by unchanged NT concentrations, determined in the serum of patients with mandibular fractures indicate that this molecule does not participate in the oxidative modification of proteins.

In addition, we have not demonstrated statistically significant differences in the levels of MDA and NT between patients depending on the number of fractures. However, it is surprising that we were able to observe lower mean concentration of MDA in the serum of patients with a single fracture than in those with two fractures and higher mean levels of NT in patients with two fractures than in those with single fracture. These observations indicate that depending on the number of injuries, other mechanisms that are responsible for maintaining the correct balance between the actions of osteoblasts and osteoclasts are activated, enabling the retention of bone durability and repairing of injuries occurring within the bone.

However, the existing data indicate that high NO concentrations may lead to the methylation of nuclear proteins through S-nitrosylation of enzymes termed protein-arginine methyltransferases (PRMTs) [36]. The product of degradation of these proteins, which is catalyzed by PRMTs, is ADMA, an endogenous inhibitor of eNOS [37,38]. The above data may explain the high ADMA concentrations observed in this study in the serum of patients with mandibular fractures before the procedure and 1 day after the procedure, as well as the correlation demonstrated between the concentrations of NO and ADMA. These results are particularly unfavorable due to the fact that high ADMA concentrations lead to endothelial dysfunction, leukocyte activation, including neutrophil degranulation, and platelet aggregation [39,40,41,42].

Furthermore, the available data show that a high level of ADMA may lead to osteoblastic dysfunction and the development of osteoporosis. Xio et al. [43] demonstrated that ADMA inhibits the NO/NOS pathway through reduced activity of alkaline phosphatases, calcium deposition, and osteoblast-related gene expression in the mouse bone morrow-derived mesenchymal cells. In a study by Kanazawa et al. [44], the high level of ADMA observed in the blood serum of patients with diabetes was linked to vertebral fractures in these individuals. The researchers suggested that the high ADMA level in this patient group may increase their risk of vertebral fractures independent of age, body height, renal function, glucose level, and occurrence of complications including neuropathy and retinopathy. In view of these data, the high ADMA concentration observed in the serum of patients with mandibular fractures before the procedure and on the first day after the procedure in the present study seems to favor excessive resorption of osteoclasts and elevated bone metabolism.

## 5. Conclusions

In summary, the course of fracture healing observed in patients with mandibular fracture at different periods was found to be associated with an increased NO concentration, as well as changes in the concentrations of MDA and ADMA, indicating that the intensity of local processes is reflected by the changes in the tested markers of the action of NO in the serum. The obtained results suggest the applicability of the determination of NO and ADMA in the assessment of mandibular fracture healing. In addition, they may constitute the basis for the development of a new group of drugs that are highly selective to the process of bone resorption, which will contribute to the NO-stimulated and ADMA-stimulated reduction of bone resorption.

## Figures and Tables

**Table 1:** Baseline characteristics of the control groups’ and patients’ blood parameters. All of the data are expressed as average ± SD. WBC—white blood cells, RBC—Red Blood Cells, HGB—Hemoglobin, HCT—Hematocrit, MCV—Mean Corpuscular Hemoglobin, MCH—Mean Cell Hemoglobin, MCHC—Corpuscular/Cellular Hemoglobin Concentration, RDW-SD—Red Cell Distribution Width Standard Deviation, RDW-CV—Red Cell Distribution Width Coefficient of Variation, PLT—Thrombocytes, PCT—Plateletcrit, MPV—Mean Platelet Volume, PDW—Platelet Distribution Width, P-LCR—Platelet Large Cell Ratio, PT—Prothrombin Time, INR—International Normalized Ratio, APTT—Activated Partial Thromboplastin Time.

	Control Group (*n* = 15)	Patients (*n* = 20)	
Parameter (Unit)	Value	Value	Reference Value
WBC (×10^3^/μL)	7.28 ± 2.5	11.16 ± 3.8	4.00–10.00
RBC (×10^6^/μL)	4.94 ± 0.21	4.83 ± 0.3	4.50–6.50
HGB (g/dL)	13.7 ± 0.5	14.93 ± 0.9	13.00–18.00
HCT (%)	45.26 ± 3.42	42.40 ± 2.3	40.00–54.00
MCV (fL)	87.04 ± 2.9	88.03 ± 3.5	82.00–94.00
MCH (pg)	29.28 ± 1.9	31.00 ± 1.4	27.00–34.00
MCHC (g/dL)	33.74 ± 0.6	35.21 ± 0.8	31.00–37.00
RDW-SD (fL)	42.39 ± 3.1	40.65 ± 4.2	37.00–47.00
RDW-CV (%)	13.81 ± 1.8	12.43 ± 1.1	11.5–15.00
PLT (×10^3^/μL)	239.82 ± 30.5	255.25 ± 44.3	130.00–350.00
PCT (%)	0.19 ± 0.06	0.26 ± 0.04	0.1–0.4
MPV (fL)	9.44 ± 1.2	10.11 ± 0.9	7.00–12.00
PDW (fL)	8.24 ± 1.7	12.30 ± 2.9	6.00–16.00
P-LCR (%)	17.35 ± 7.3	25.23 ± 8.0	6.00–40.00
PT (sec.)	12.81 ± 0.9	12.39 ± 0.6	11.50–15.00
INR (sec.)	0.83 ± 0.05	0.95 ± 0.04	0.8–1.2
APTT (sec.)	27.34 ± 2.53	28.79 ± 3.2	24.00–35.00
APTT Ratio	0.99 ± 0.18	0.96 ± 0.1	0.8–1.2
Sodium (mmol/L)	139.72 ± 3.1	137.82 ± 2.4	136.0–145.0
Potassium (mmol/L)	3.9 ± 0.33	4.60 ± 0.4	3.5–5.1

**Table 2:** Concentrations of total NO, MDA, NT, and ADMA in the serum of patients respect to the number of fractures within the mandible.

		NO Mean (±SD)	MDA Mean (±SD)	NT Mean (±SD)	ADMA Mean (±SD)
**Control**		55.19 (±12.24)	356.21 (±122.11)	482.42 (±218.6)	166.93 (±59.15)
T0	Single fracture	90.14 * (±21.52)	222.58 * (±85.41)	407.28 (±101)	286.43 * (±142.6)
	Double fracture	78.71 * (±22.39)	208.82 * (±44.41)	540.27 (±182.22)	259.67 * (±74.22)
T1	Single fracture	96.58 * (±30.61)	233.35 * (±77.8)	486.91 (±156.85)	255.19 * (±73.15)
	Double fracture	79.97 * (±20.07)	162.9 * (±45.48)	582.65 (±207.31)	262.11 * (±106.49)
T2	Single fracture	102.17 * (±19.21)	295.51 (±32.76)	421.85 (±247.32)	124.36 (±58.95)
	Double fracture	76.65 (±27.96)	207.7 * (±76.72)	629.2 (±236.82)	164.24 (±81.96)
T3	Single fracture	88.59 * (±6.15)	191.73 * (±34.45)	416.33 (±170,43)	129.04 (±36.87)
	Double fracture	86.41 * (±5.52)	184.11 * (±25.05)	787.51 (±189.25)	83.16 (±21.36)

All data are expressed as mean ± SD. Patients group: before surgery (T0), at 24 h after surgery (T1), at 2 weeks (T2), and 6 weeks (T3) after surgery. *—Statistical differences with control.

## Data Availability

The datasets used and/or analyzed during the current study are available from the corresponding author on reasonable request.

## Abbreviations

NO: total nitric oxide; MDA: malonyldialdehyde, NT: nitrotyrosine, ADMA: asymmetric dimethylarginine; iNOS: inducible nitric oxide synthase; eNOS: endothelial nitric oxide synthase SD: standard deviation.
